# MLLT11/AF1q boosts oncogenic STAT3 activity through *Src*-PDGFR tyrosine kinase signaling

**DOI:** 10.18632/oncotarget.9759

**Published:** 2016-06-01

**Authors:** Jino Park, Soojin Kim, Joongho Joh, Scot C. Remick, Donald M. Miller, Jun Yan, Zeyad Kanaan, Ju-Hsien Chao, Maxwell M. Krem, Soumit K. Basu, Shotaro Hagiwara, Lukas Kenner, Richard Moriggl, Kevin D. Bunting, William Tse

**Affiliations:** ^1^ James Graham Brown Cancer Center, University of Louisville School of Medicine, Louisville, KY, USA; ^2^ Division of Blood and Bone Marrow Transplantation, Department of Medicine, University of Louisville School of Medicine, Louisville, KY, USA; ^3^ Maine Medical Center Research Institute, Portland, ME, USA; ^4^ Department of Medicine and Department of Microbiology and Immunology, University of Louisville, Louisville, KY, USA; ^5^ Division of Hematology, Internal Medicine, National Center for Global Health and Medicine, Shinjuku, Japan; ^6^ Ludwig Boltzmann Institute for Cancer Research, Vienna, Austria; ^7^ Clinical Institute for Pathology, Medical University of Vienna, Vienna, Austria; ^8^ Unit of Pathology of Laboratory Animals (UPLA), University of Veterinary Medicine, Vienna, Austria; ^9^ Institute of Animal Breeding and Genetics, University of Veterinary Medicine Vienna, Medical University of Vienna, Vienna, Austria; ^10^ Aflac Cancer and Blood Disorders Center of Children's Healthcare of Atlanta and Emory University School of Medicine, Atlanta, GA, USA

**Keywords:** AF1q, STAT3, PDGF, PDGFR, Src

## Abstract

Constitutive STAT3 activation by tyrosine phosphorylation of mutated or amplified tyrosine kinases (pYSTAT3) is critical for cancer initiation, progression, invasion, and motility of carcinoma cells. We showed that AF1q is associated with STAT3 signaling in breast cancer cells. In xenograft models, enhanced AF1q expression activated STAT3 and promoted tumor growth and metastasis in immunodeficient NSG mice. The cytokine secretory phenotype of MDA-MB-231LN breast cancer cells with altered AF1q expression revealed changes in expression of platelet-derived growth factor subunit B (PDGF-B). AF1q-induced PDGF-B stimulated motility, migration, and invasion of MDA-MB-231LN cells, and AF1q up-regulated platelet-derived growth factor receptor (PDGFR) signaling. Further, AF1q-induced PDGFR signaling enhanced STAT3 activity through *Src* kinase activation, which could be blocked by the *Src* kinase inhibitor PP1. Moreover, AF1q up-regulated tyrosine kinase signaling through PDGFR signaling, which was blockable by imatinib. In conclusion, we demonstrated that enhanced AF1q expression contributes to persistent and oncogenic pYSTAT3 levels in invasive carcinoma cells by activating *Src* kinase through activation of the PDGF-B/PDGFR cascade. Therefore, AF1q plays an essential role as a cofactor in PDGF-B-driven STAT3 signaling.

## INTRODUCTION

Signal transducer and activator of transcription 3 (STAT3) is a critical signaling intermediate for many important cellular and biological processes in carcinogenesis, including inflammation, cellular transformation, proliferation, survival, invasion, angiogenesis, and metastasis [1–3]. Aberrant STAT3 signaling has been implicated in carcinogenesis: STAT3 is constitutively activated in cells transformed by diverse oncoproteins or tumor viruses, and elevated STAT3 signaling prevents apoptosis and confers survival advantages like chemo-resistance or radio-resistance [4]. STAT3 can be activated by non-receptor-associated kinases (eg, Src) or receptor-associated kinases (eg, JAK) via phosphorylation of tyrosine 705 (pYSTAT3). STAT3 tyrosine 705 phosphorylation can occur by stimulation of growth factor receptors with tyrosine kinase activity (eg, epidermal growth factor receptor [EGFR], platelet-derived growth factor receptor [PDGFR], hepatocyte growth factor receptor [HGFR or cMET], as well as cytokine receptors lacking intrinsic tyrosine kinase activity (eg, gp130 cytokine family) [5–9]. Activated STAT3 forms homo- or heterodimers with STAT1 through reciprocal *Src* Homology 2 (SH2)-pTyr interactions. The two known splice forms of STAT3 α/β differ in their transactivation domain and DNA binding affinity, which impacts also pYSTAT3 stability. Tyrosine phosphorylation of STAT3 results in translocation of STAT3 to the nucleus, where it regulates expression of target genes harboring STAT3 binding sites in their transcriptional regulatory region [10].

STAT3 can also be phosphorylated on a critical serine residue at position S727, used by the H-Ras oncoprotein and activated for example by the MEK-ERK pathway, essential for mitochondrial STAT3 traffic and function [11]. Mitochondrial serine-phosphorylated STAT3 is essential for RAS transformation via control of oxidative phosphorylation, which has an impact on overall reactive oxygen species (ROS) production and energy supply. ROS production is involved in cancer stem cell renewal, differentiation of epithelial cells, DNA double strand breaks and repair processes, lipid or protein oxidation, and inactivation of the catalytic center of tyrosine phosphatases prolonging the action of the JAK-STAT pathway [12, 13].

Moreover, STAT3 expression has been shown to correlate with PDGF-B expression, a well-described initiator of brain cancer. STAT3 activation alone was insufficient to induce brain cancer formation, but co-expression of STAT3 with PDGF-B in a transgenic mouse model resulted in efficient glioma multiforme formation [14]. PDGF promotes cell migration, proliferation and survival by binding to its cognate tyrosine kinase receptor PDGFR, which consists of α and β chains [15]. The homodimer PDGF-BB is the only PDGF that can bind both homo- and heterodimers of PDGFR with high affinity [16]. Also, Src kinase activation has been reported to contribute to PDGF-dependent cell-cycle progression, mitogenesis, and chemotaxis through its association with PDGFR-β in vitro [17, 18].

Our lab originally identified AF1q as an MLL fusion partner in acute myeloid leukemia patients with a t(1; 11)(q21; q23) translocation. We demonstrated that AF1q expression is associated with poor clinical outcomes in myeloid malignancies, and a number of studies have shown that AF1q plays a role in lung and breast cancer metastasis [19–23]. However, other reports indicated that AF1q could also influence pro-apoptotic effects mediated by BAD or fenretinide-induced ROS production [24, 25]. The consensus today is that AF1q plays an important role in malignancy of solid tumors, but the molecular mechanisms by which AF1q interacts with oncoproteins or influences tumor suppressor gene loss are incompletely understood. We previously demonstrated that AF1q physically interacts with the HMG box protein TCF7, a key factor in Wnt signaling, and AF1q enhances expression of Wnt target genes [26]. The AF1q-TCF7 interaction results in enhanced expression of CD44, a ubiquitous multi-structural and multi-functional cell surface glycoprotein involved in adhesion, migration, and homing of cells. We also demonstrated that elevated AF1q expression is significantly associated with breast cancer tumorigenesis and metastasis using patient-derived analysis and in vivo xenograft mouse models, combined with paired breast cancer cell line studies with enforced or suppressed AF1q.

In our earlier studies, we observed that STAT3 is activated when AF1q is expressed in breast cancer cells. We questioned whether the STAT3 pathway is influenced by AF1q expression, because in colorectal cancer progression it was convincingly shown that both STAT3 and Wnt signaling are needed for full malignancy and cancer progression [27]. To date, the mechanism of STAT3 activation by AF1q has not been studied. Here, we investigate how AF1q induces the activation of STAT3, and whether AF1q-induced STAT3 activation involves *Src*kinase and PDGFR signaling.

## RESULTS

### Enhancing AF1q expression activates STAT3 and promotes tumor growth and metastasis in immunodeficient Nod/Scid/Gamma chain null (NSG) mice

**Figure 1:**
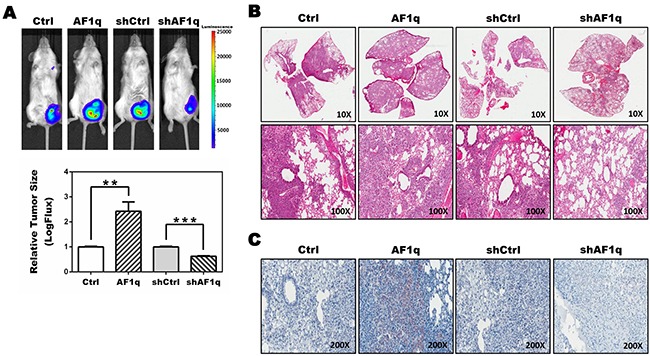
NSG xenograft mouse models demonstrate an association between AF1q expression and STAT3 activation **A.** Representative images of NSG mice (n=5) 4 weeks after injection with MDA-MB-231LN/Ctrl, /AF1q, /shCtrl or /shAF1q cells in mammary fat pads (left panel). Quantification was carried out by measuring tumor size (right panel). Tumor size is shown as mean ± SD. *P* values were calculated using a student *t* test (*, *P*<0.05; **, *P*<0.01; ***, *P*<0.001). **B.** The lungs were harvested from NSG xenograft mouse models at 6 weeks after injection. MDA-MB-231LN/AF1q injected mice grew significantly larger metastasis tumor masses compared to /Ctrl mice. Suppressed the endogenous AF1q expression with shRNA in MDA-MB-231LN were significantly smaller tumors. **C.** Formalin-fixed sections of lung metastasis nodules from NSG xenograft mouse models were evaluated by immunohistochemistry for pYSTAT3 expression.

We recently demonstrated the contribution of AF1q to breast carcinoma formation *in vivo* using xenograft models of paired human breast cancer cell lines with enforced or suppressed AF1q expression injected into immunodeficient NSG mice [26]. We used the same system in this study to determine the mechanistic impact of AF1q expression on breast cancer progression. MDA-MB-231LN breast cancer cells stably expressing AF1q (MDA-MB-231LN/AF1q) or empty vector-transduced cells (MDA-MB-231LN/Ctrl) were injected into mammary fat pads of NSG mice. For the loss assay, MDA-MB-231LN cells stably expressing shRNA against AF1q (MDA-MB-231LN/shAF1q) and scramble-transduced cells (MDA-MB-231LN/shCtrl) were used. MDA-MB-231LN/AF1q-injected mice had significantly more aggressive tumor growth in mammary fat pads and metastasis compared with MDA-MB-231LN/Ctrl-injected mice, while MDA-MB-231LN/shAF1q-injected mice had attenuated tumor growth and metastasis compared with MDA-MB-231LN/shCtrl-injected mice (Figure 1A, 1B and Supplementary Figure S1), in line with our published results [26]. Previously we showed that AF1q plays a critical role in breast cancer initiation, progression, and metastasis through activation of the Wnt signaling pathway [26]. Entangled crosstalk between Wnt and other signaling pathways (i.e. Notch and STAT3) triggered other signaling pathways involved in cancer initiation, progression, and metastasis [28, 29]. Thus, alterations in Wnt signaling can lead to a variety of disorders, including cancer. Among signaling pathways which crosstalk with the Wnt signaling pathway, STAT3 activation has been particularly linked with breast cancer progression [30–34]. To evaluate STAT3 activation, tissues from our xenograft model were isolated, sectioned, and stained with anti-pYSTAT3 4 weeks post-injection. Lung metastasis nodules from MDA-MB-231LN/AF1q-injected mice had very strongly activated pYSTAT3, whereas normal lung and lung metastasis nodules from MDA-MB-231LN/shAF1q-injected mice did not (Figure 1C). Collectively, enforced AF1q expression promoted increased tumor growth and metastasis associated with elevated pYSTAT3 induction.

### AF1q regulates PDGF-B expression through activation of its transcriptional pathway

**Figure 2:**
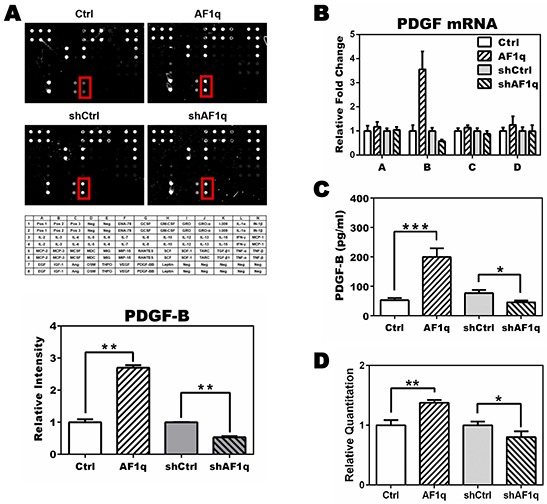
PDGF-B expression is altered by AF1q expression **A.** The human cytokine array G3 (RayBiotech) was used to detect 42 human cytokines in lysate from MDA-MB-231LN/Ctrl, /AF1q, /shCtrl, and /shAF1q cells. PDGF-B expression, indicated by the red rectangle in each panel, is quantified in the graph below. Mean expression in /Ctrl cells and /shCtrl cells was normalized to 1. The intensity of /AF1q was compared with /Ctrl and the intensity of /shAF1q was compared with /shCtrl, because those paired groups used different backbone viral vectors, pLUTdNB and pTRIPZ, respectively. Bars indicate the SD. The table shows each target protein on the array slides. **B.** mRNA expression of the four members of the PDGF family (A, B, C, D) was determined by qPCR. Enforced AF1q increased the expression of PDGF-B mRNA (~3.5 fold) and suppressed AF1q expression using shRNA attenuated PDGF-B mRNA expression by 2 fold. Only PDGF-B was regulated by AF1q. **C.** The culture medium was harvested 24 h after doxycycline treatment to induce AF1q or shAF1q expression, and subjected to quantitative ELISA to assess PDGF-B protein concentration. **D.** Luciferase activity in MDA-MB-231LN cells transfected with reporter constructs of the proximal promoter of PDGF-B. *Firefly* luciferase was normalized to *Renillar* luciferase activity. Values are means ± SD of three independent experiments. *P* values were calculated using a student *t* test (*, *P*<0.05; **, *P*<0.01; ***, *P*<0.001).

To investigate how AF1q expression alters STAT3 activation, we examined the expression of cytokines, growth factors, and chemokines in MDA-MB-231LN/Ctrl, /AF1q, /shCtrl, and /shAF1q cells using a human cytokine array. We found that PDGF-B was differentially regulated in response to AF1q expression (Figure 2A). To confirm whether PDGF-B mRNA and protein expression were elevated, we used mRNA qPCR and ELISA, respectively, in each group of MDA-MB-231LN cells and cultured medium. We determined that the concentration of PDGF-B increased about three-fold when AF1q expression was enforced and decreased about two-fold when AF1q expression was suppressed (Figure 2B-2C). To investigate the mechanism underlying enhanced PDGF-B expression, we evaluated the activity of the PDGF-B promoter, determined as the ratio of the activity of *firefly* luciferase to the internal control *Renilla* luciferase. Enforced AF1q expression activated the PDGF-B promoter, while suppressed AF1q expression attenuated the activity of the PDGF-B promoter (Figure 2D).

### AF1q-induced PDGF-B stimulates the migration and invasion of breast cancer cells

**Figure 3:**
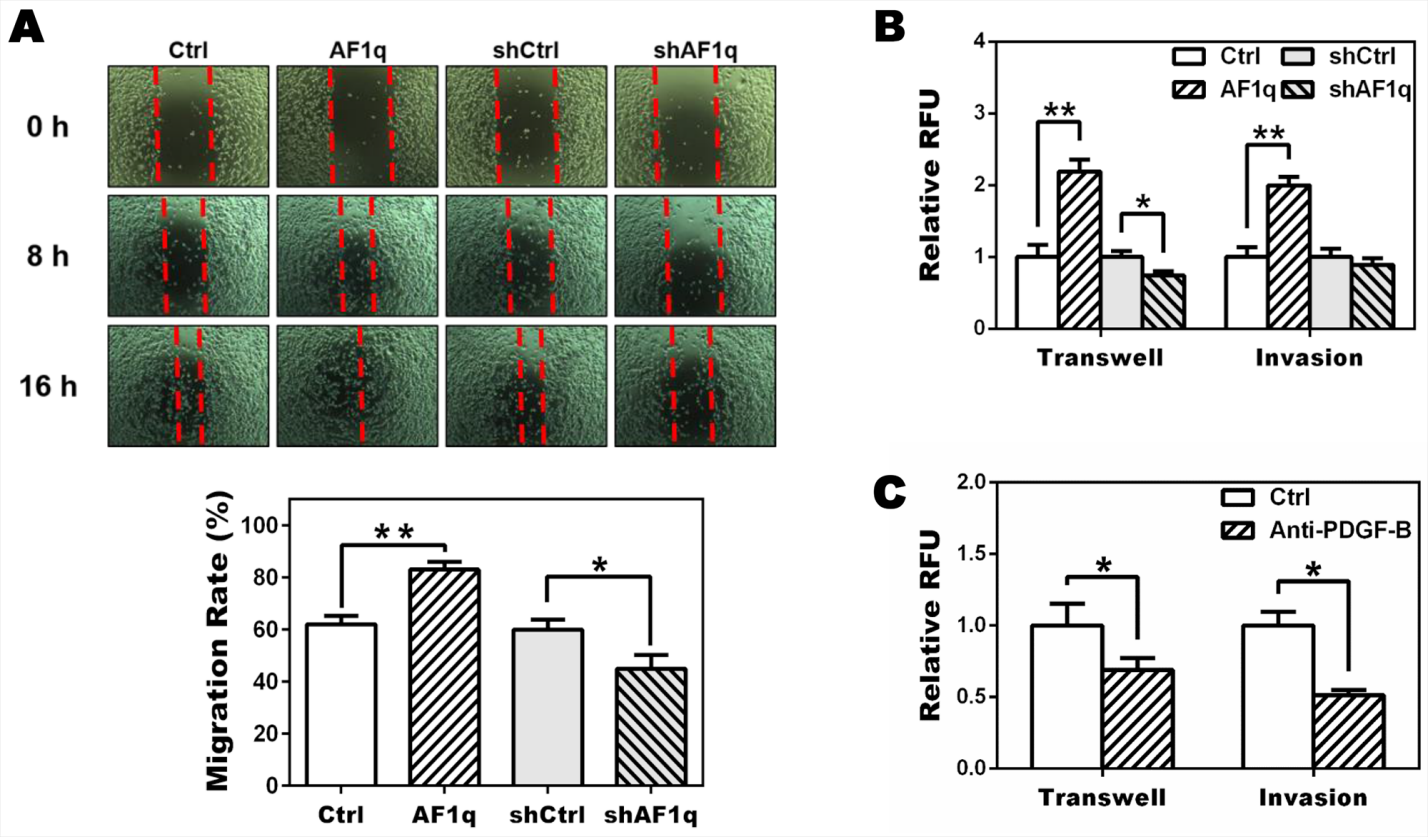
Targeting PDGF-B inhibits migration of MDA-MB-231LN cells **A.** A wound healing assay was performed to determine the effect of AF1q-induced PDGF-B on motility of MDA-MB-231LN cells. Cell monolayers were wounded with a micropipette tip, washed twice with culture medium and cultured with conditioned medium (CM) from MDA-MB-231LN/Ctrl, /AF1q, /shCtrl, and /shAF1q cells, respectively, and then photographed at 0, 8 and 16 h. Quantification was carried out by measuring distance between the borders (bottom panel). **B.** The CM from MDA-MB-231LN/AF1q cells promoted transwell migration and matrigel invasion compared with CM from their corresponding controls (Ctrl), whereas CM from MDA-MB-231LN/shAF1q cells decreased migration and invasion of MDA-MB-231LN cells. **C.** MDA-MB-231LN cells were incubated with CM from MDA-MB-231LN/AF1q cells in the presence of a neutralizing PDGF-B antibody (5 μg/ml) or isotype antibody (5 μg/ml). Cell migration and invasion were attenuated by the PDGF-B antibody. *P* values were calculated using a student *t* test (*, *P*<0.05; **, *P*<0.01; ***, *P*<0.001).

To investigate whether AF1q-induced PDGF-B altered the behavior of MDA-MB-231LN cells, MDA-MB-231LN cells were incubated with conditioned medium (CM) from MDA-MB-231LN/Ctrl, /AF1q, /shCtrl, and /shAF1q cells, and levels of cell migration and invasion were examined using wound healing, transwell migration, and matrigel invasion assays, respectively. As shown in Figure 3A-3B, CM from MDA-MB-231LN/AF1q cells stimulated motility, migration and invasion of MDA-MB-231LN cells compared with controls, whereas CM from MDA-MB-231LN/shAF1q cells decreased migration and invasion of MDA-MB-231LN cells.

To verify these findings, we used an antibody to neutralize AF1q-induced PDGF-B in CM from MDA-MB-231LN/AF1q cells and performed the transwell migration and matrigel invasion assay. As shown in Figure 3C, when AF1q-induced PDGF-B was neutralized, migration and invasion of MDA-MB-231LN cells was reduced. Taken together, these results show that AF1q expression in MDA-MB-231LN cells has paracrine activity through PDGF-B to promote motility, migration and invasion of MDA-MB-231LN cells.

### AF1q activates STAT3 through PDGF-B/PDGFR tyrosine kinase signaling

**Figure 4:**
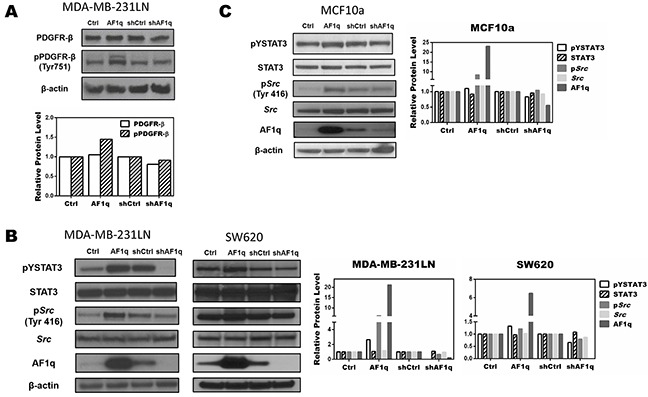
AF1q controls STAT3 activation by modulating Src phosphorylation through the PDGF-B/PDGFR signaling cascade **A.** AF1q induced PDGFR-β phosphorylation in MDA-MB-231LN cells, as determined by Western blot. **B.** Constitutive expression and phosphorylation of *Src* and STAT3 protein in MDA-MB-231LN and SW620 cells transformed to enforce or suppress AF1q. **C.** Constitutive phosphorylation and expression levels of *Src* and STAT3 protein in normal breast epithelial MCF10a cells after transformation to enforce or suppress AF1q.Band density was determined by ImageJ analysis software.

To investigate further the mechanism underlying AF1q-induced PDGF-B, we evaluated the PDGF/PDGFR signaling pathway in MDA-MB-231LN cells using Western blot. Up-regulating PDGF-B through an AF1q-dependent transcriptional mechanism induced tyrosine phosphorylation of PDGFR in MDA-MB-231LN cells. Conversely, suppressing AF1q expression by shAF1q effectively down-regulated activation of the PDGF/PDGFR signaling pathway. These results indicate that PDGF-B/PDGFR signaling was up-regulated by AF1q (Figure 4A). To investigate the mechanism responsible for promoting migration and invasion, we examined whether signaling downstream from PDGF-B/PDFGR was altered in two carcinoma cell lines, MDA-MB-231LN breast cancer cells and SW620 colon cancer cells, transformed to enforce or suppress AF1q. It has been shown that constitutive STAT3 activity in both breast and colon cancer contributes to enhanced cell proliferation and metastasis [35, 36]. As shown in Figure 4B, AF1q-induced PDGF-B/PDGFR signaling effectively induced *Src* kinase activation, as measured with Y416 phospho-specific *Src* antibody Western. Then, we evaluated STAT3 expression and activation status. Interestingly, pYSTAT3 was strongly induced and activated in cells with enforced AF1q expression. Because MDA-MB-231LN breast cancer cell lines are known to possess constitutively activated *Src* kinases, we examined STAT3 phosphorylation levels in MCF10a normal human breast epithelial cells with enforced or suppressed AF1q expression. As shown in Figure 4C, pYSTAT3 and p*Src* were effectively increased in MCF10a cells with enforced AF1q expression, but attenuated when AF1q expression was suppressed.

### STAT3 activation by AF1q requires the phosphorylation of *Src* kinase

**Figure 5:**
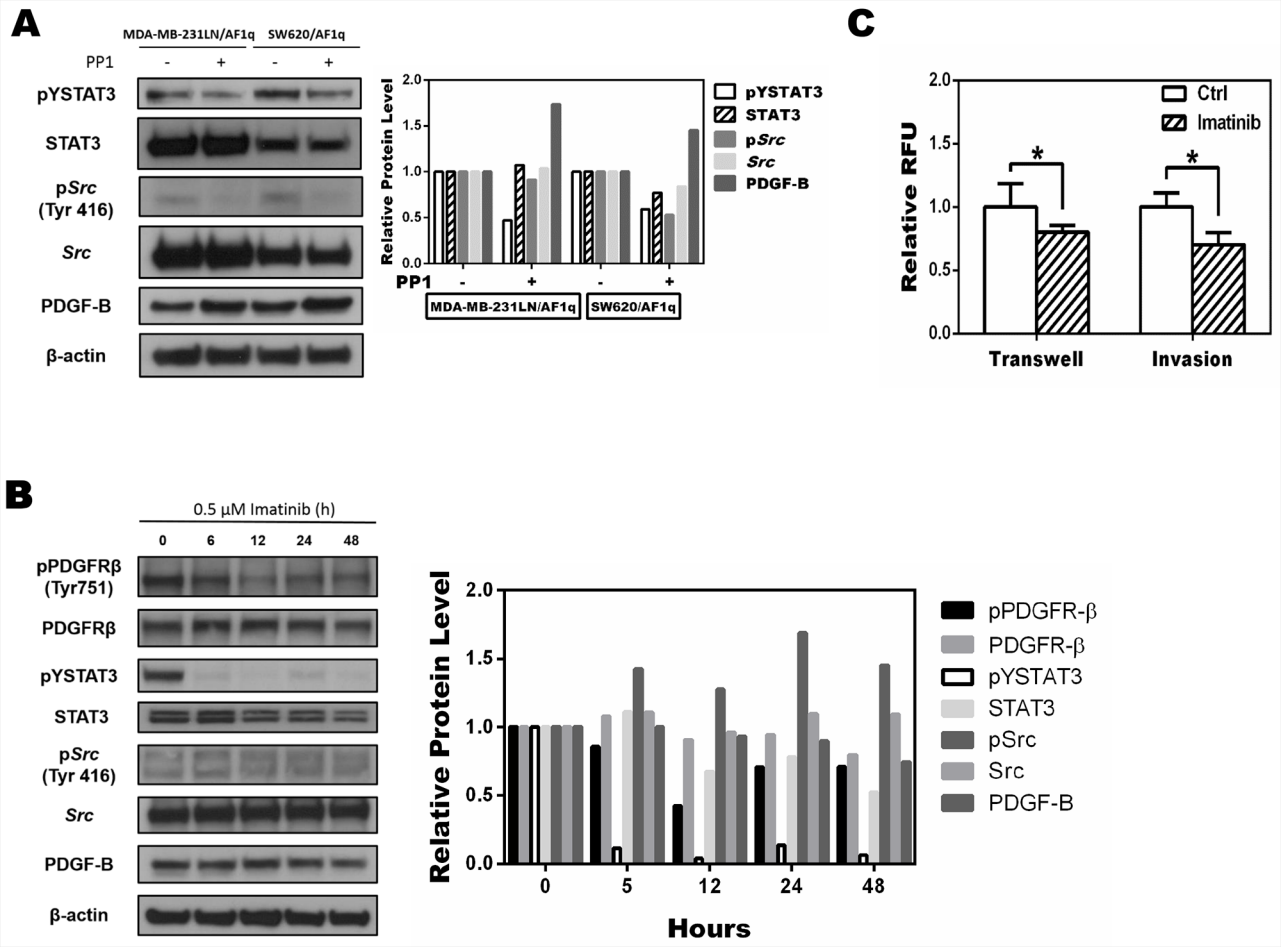
Targeting the phosphorylation of Src inhibits AF1q-induced STAT3 activation **A.** *Src* phosphorylation of MDA-MB-231LN/AF1q and SW620/AF1q cells was inhibited by PP1, a selective *Src* inhibitor, and pYSTAT3 expression also decreased. **B.** MDA-MB-231LN/AF1q cells were treated with imatinib (0.5 μM) and expression of PDGFR-β, STAT3, *Src*, and PDGF-B were assessed by Western blot 0, 6, 12, 24, and 48 h later. Band density was determined by ImageJ analysis software. **C.** Quantification of transwell migration and invasion of MDA-MB-231LN/AF1q after imatinib treatment for 24h. *P* values were calculated using a student *t* test (*, *P*<0.05; **, *P*<0.01; ***, *P*<0.001).

*Src* has been shown to directly increase pYSTAT3 levels [37, 38]. To evaluate whether *Src* was responsible for AF1q-induced STAT3 activation, we treated MDA-MB-231LN/AF1q and SW620/AF1q cells with 0.1 μM of the selective *Src* inhibitor PP1 for 24 h [39]. The phosphorylation of *Src* decreased significantly, and STAT3 phosphorylation also decreased (Figure 5A). Conversely, suppressing AF1q expression by shAF1q effectively down-regulated activation of the PDGF/PDGFR signaling pathway. These results indicate that AF1q-induced PDGF-B/PDGFR signaling triggered the activation of STAT3 by *Src* kinase.

Imatinib, a FDA-approved tyrosine kinase inhibitor specific for oncogenic fusions or mutations of PDGFRs, c-KIT, and ABL tyrosine kinase oncogenes, has been an effective or even curative treatment for certain leukemias [40]. We applied imatinib (0.5 μM) to MDA-MB-231LN/AF1q cells and evaluated the activity status of key proteins associated with PDGF/PDGFR signaling using Western blot. Phosphorylation of pYPDGFR-β, pYSTAT3, and pY*Src* in MDA-MB-231LN/AF1q cells was inhibited by imatinib, but PDGF-B expression was not changed (Figure 5B). Notably, imatinib inhibited the migration and invasion of MDA-MB-231LN/AF1q cells (Figure 5C). Our results indicate that imatinib exerts a significant inhibitory effect on breast cancer cell invasion and migration through inhibition of PDGFR and STAT3 phosphorylation, although the expression of PDGF-B was unchanged. This result is in line with data we observed with the anti-PDGF-B inhibitory antibody (Figure 3C). Collectively, our data suggest that the AF1q-induced PDGF-B/PDGFR signaling cascade is directly associated with altered cell invasion and migration.

### AF1q-induced STAT3 activation via *Src*-PDGFR tyrosine kinase signaling increases binding activity

**Figure 6:**
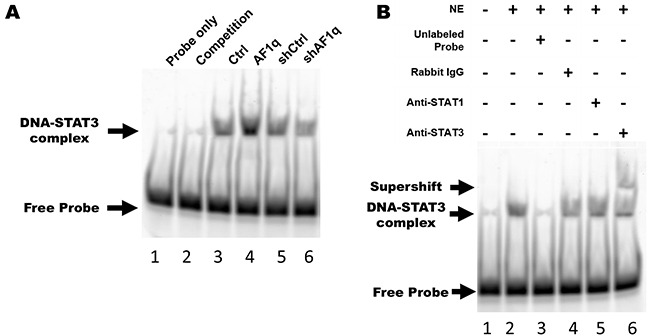
AF1q-induced PDGF-B enhances STAT3 binding activity through the PDGFR signaling cascade **A.** Nuclear protein was isolated from MDA-MB-231LN/Ctrl, /AF1q, /shCtrl, and /shAF1q cells, and EMSA was performed using Alexa 488-labeled STAT3 hSIE probes. Lane 1, free labeled probes without nuclear extract (negative control); lane 2, labeled probes and 10 fold molar excess unlabeled probes (competitor) with nuclear extract of MDA-MB-231/Ctrl; lane 3-6, labeled probe with nuclear extract from each cell type. The upper arrow points to the position of the STAT3-hSIE probe complex. The intensity of this band is proportional to the amount of STAT3 translocated to the nucleus and bound to DNA. **B.** Supershift assay using MDA-MB-231LN/AF1q nuclear extracts revealed that STAT3 bound to probe, not STAT1. Lane 1, free labeled probes without nuclear extract (NE); lane 2, labeled probes with NE; lane 3, labeled probes and 10 fold molar excess unlabeled probes (competitor) with nuclear extract; lane 4, labeled probes and isotype rabbit IgG antibody with nuclear extract; lane 5, labeled probes and anti-STAT1 antibody with nuclear extract; lane 6, labeled probes and anti-STAT3 antibody with nuclear extract.

To verify whether an increase in AF1q-induced STAT3 phosphorylation was accompanied by an increase in STAT3 binding activity, we performed electrophoretic mobility shift assays (EMSA) using a hSIE oligonucleotide probe. The oligonucleotide Alexa 488-labeled probe was incubated with nuclear protein from MDA-MB-231LN cells. The nuclear protein formed a protein-DNA complex with the hSIE oligonucleotide, but no protein-DNA complex formed with the competitor hSIE oligonucleotide or when nuclear protein extract was omitted. In addition, band intensity reflects the level of DNA binding activity in response to STAT3 phosphorylation. As shown in Figure 6A, we found that enforced AF1q expression in MDA-MB-231LN cells enhanced the binding affinity of STAT3 for the hSIE oligonucleotide probe. Conversely, the binding affinity of STAT3 was decreased when AF1q expression was suppressed in MDA-MB-231LN cells. Moreover, a specific C-terminal STAT3 antibody caused gel retardation of the hSIE-containing complexes in a supershift assay, but a STAT1 antibody did not cause severe gel retardation (Figure 6B). Taken together, the PDGF-B/PDGFR signaling cascade was activated upon enforced AF1q expression and this caused an increase in STAT3 DNA binding activity through *Src* kinase action in MDA-MB-231LN cells.

## DISCUSSION

**Figure 7:**
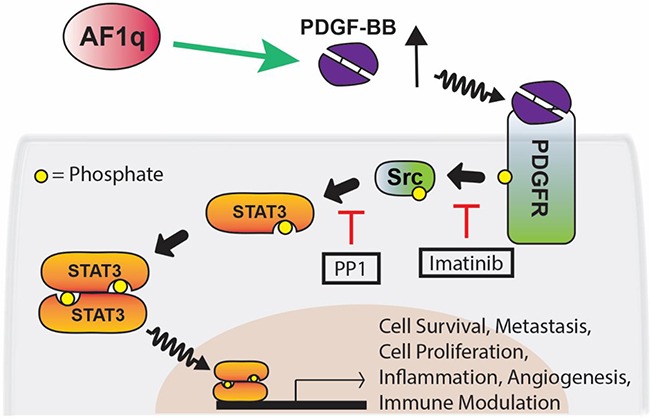
Proposed network for AF1q-induced PDGF-B/PFGFR/Src/STAT3 signaling AF1q enhances the expression of PDGF-B, which forms homodimers (PDGF-BB) that bind to the protein tyrosine kinase receptor PDGFR. Binding of PDGF-BB to PDGFR results in tyrosine phosphorylation of PDGFR. *Src* is recruited to phosphorated PDGFR and activated by phosphorylation of tyrosine 416. Activation of STAT3 occurs via phosphorylation of tyrosine 705 through the *Src* tyrosine kinase. Using selective inhibitors of tyrosine kinases (PP1 and imatinib), we showed that PDGFR and *Src* cooperate to mediate STAT3 activation in breast cancer cell lines. This signaling pathway affects multiple cellular processes including cell survival, metastasis, cell proliferation, inflammation, angiogenesis, and immune modulation. Green arrows and red lines indicate activation and suppression, respectively.

STAT3 is constitutively activated in many human cancers with poor prognosis or chemo- or radio-resistance, which have been molecularly linked through cancer genome landscape studies to increased tyrosine kinase signaling in particular [41–43]. Aberrant STAT3 signaling is an important process in malignant progression. Hence, greater understanding of the regulatory mechanism of STAT3 is important to improve treatment. We found that AF1q activates STAT3 in human breast cancer cells by activating *Src* kinase through activation of the PDGF-B/PDGFR cascade. The transcriptionally amplified PDGF-B regulated by AF1q accelerated activation of the PDGFR-*Src* kinase cascade, driving persistent STAT3 activation (pYSTAT3) in breast cancer cells (Figure 7). A number of reports have suggested that phosphorylation of serine 727 (pSSTAT3) promotes breast cancer growth by localizing to mitochondria [44]. We tested whether pSSTAT3 is also activated by AF1q using western blot. We found that pSSTAT3 was not changed by expression of AF1q (Supplementary Figure S2).

We observed that MDA-MB-231LN/AF1q breast cancer cells show about 2.5-fold more aggressive tumor growth (Figure 1A) and approximately 4 times the number of lung metastatic colonies (data not shown) than control cells in NSG mice. Also, we observed that pYSTAT3 levels were significantly elevated in lung metastatic colonies (Figure 1B). We also provided mechanistic insight into the effects of AF1q-induced STAT3 activation at the cellular level. To explain these observations, we investigated kinase signaling upstream of the STAT3 signaling pathway and proved that AF1q activated the 1,095 bp promoter region of PDGF-B, a growth factor ligand that binds to PDGFR to enhance autoregulation and stimulation of PDGFR signaling. In the PDGF family, only PDGF-B was associated with AF1q. To identify the mechanism of enhanced PDGF-B expression induced by AF1q, we performed aPDGF-B promoter assay using the luciferase reporter system. It is clear that AF1q boosts PDGF-B expression through activation of the PDGF-B promoter region. We next examined whether PDGF-B regulates cell migration. PDGF and PDGFR may participate in cellular migration and PDGF-B is the strongest inducer in that process [45–47]. As expected, PDGF-B regulated cell migration and we confirmed this finding by an inhibition assay. Our studies showed that imatinib inhibited migration of MDA-MB-231LN cells (Figure 5C). One interpretation of these results is that disruption of PDGFR-β/PDGF-B signaling may be important to block activation of STAT3 signaling, due to the effects of imatinib on inhibition of PDGFR phosphorylation in breast cancer (Figure 5B) [48].

We also investigated whether PDGF-B promoted phosphorylation of PDGFR-α and PDGFR-β by Western blot. However, we detected a PDGF-B-dependent increase in the phosphorylation of PDGFR-β only. Repeated attempts failed to detect significant phosphorylation of PDGFR-α, which could have been due to problems with the antibodies we used or to exceptionally low PDGFR- α levels.

Because activation of STAT3 occurs in cancers with activated *Src* kinase, we examined regulation of *Src* kinase in MDA-MB-231 cells overexpressing or suppressing AF1q. We observed a cooperative activation of *Src* kinase with AF1q expression in MDA-MB-231LN cells, which correlates with cooperative oncogenic signaling by kinases in breast cancer. Furthermore, we observed that the activation of STAT3 was inhibited by PP1, a selective *Src* kinase inhibitor, in MDA-MB-231LN cells overexpressing AF1q (Figure 5A). These results demonstrate a fundamental mechanistic regulation of STAT3 by AF1q. Using EMSA analysis, we also determined that constitutive activation of STAT3 DNA-binding activity was enhanced by AF1q expression (Figure 6).

We earlier showed that the coactivator peptide AF1q activates Wnt signaling via direct interaction with TCF7, forming a transcriptional complex in conjunction with LEF1 and β-catenin [26]. Other groups have shown that the gp130-Jak-STAT3 signaling pathway promotes intestinal tumor growth and regeneration [27], as does Wnt signaling. Interestingly, the tumor growth and regeneration mediated by the Wnt signaling pathway can be prevented by inhibition of gp130-Jak-STAT3 signaling [27]. However, it was not clear if these two pathways share any common components, or how they interact. Here, we showed that inhibition of AF1q-induced PDGF-B limits the migration response of breast carcinoma cells. Thus, AF1q is a more general cofactor for both Wnt and STAT signaling, with the latter activated through the *Src*-PDGFR kinase cascade. In both cases, binding of AF1q to transcription factors results in higher transcriptional rates of genes required for tumorigenesis or metastasis. Thus, AF1q is an acidic cofactor peptide that boosts the transactivation potential of proto-oncogene transcription factors associated with invasive properties of cancer cells.

We earlier demonstrated that AF1q-positive cancer cells have significantly greater prevalence at metastatic sites than in primary breast tumors [26]. Together with our current results, these observations suggest that AF1q-positive breast cancer cells might contain persistently high levels of pYSTAT3 due to high levels of PDGF-B, which translates into a migration advantage. To investigate that possibility, we plan to evaluate AF1q, pYSTAT3, and PDGF-B expression in paired human samples of primary breast tumors and matched metastatic cancers.

In summary, our results suggest that inhibition of AF1q-induced PDGF-B might hold therapeutic promise for metastatic breast cancer. Our studies here have addressed for the first time the impact of AF1q on PDGF-B-driven STAT3 signaling.

## MATERIALS AND METHODS

### Cell culture

MCF10a and SW620 cells were purchased from the American Type Culture Collection (ATCC). MDA-MB-231-luc-D3H2LN (MDA-MB-231LN) cells were purchased from Caliper Life Science. MCF10a cells were maintained in DMEM/F-12 (1:1) medium (Invitrogen) supplemented with 5% horse serum (Gibco), 0.5 μg/ml hydrocortisone, 10 μg/ml insulin, 20 ng/ml epidermal growth factor, and 0.1 μg/ml cholera endotoxin (Sigma-Aldrich). MDA-MB-231LN and SW620 cells were cultured in DMEM medium (Invitrogen) supplemented with 10% fetal bovine serum (FBS, Gibco). They were cultured at 37°C in a 5% CO_2_ humidified atmosphere.

### Viral production and infection

Replication of incomplete lentiviral particles was performed by co-transfecting three plasmids into HEK293T cells (ATCC) with the lentiviral constructs pVSV-G and psPAX2 (Addgene) using Lipofectamine 2000 (Invitrogen). Lentivirus in the supernatant was harvested 48, 72, and 96 h after transfection and concentrated by 10% PEG-8000 (Sigma-Aldrich). Cells were transduced with concentrated lentivirus using 8 μg/ml Polybrene (Santa Cruz). Cells which underwent lentiviral transduction were selected using 1 μg/ml puromycin (Thermo Fisher) for 1 week as previously described [49]. After antibiotic selection, cells were cultured in complete medium including 1 μg/ml doxycycline (Thermo Fisher) for AF1q or shRNA induction. The control lentiviral vector for pLUTdNB-AF1q, designated Ctrl, was pLUTdNB empty vector, while pTRIPZ-Scramble, designated shCtrl, stably expressed scramble (GE Dharmacon). In this study, all cells were divided into four groups, designated MDA-MB-231LN/Ctrl, /AF1q, /shCtrl or /shAF1q.

### Measurement of cytokine, chemokine, and growth factor production

Expression of cytokines, chemokines, and growth factors by MDA-MB-231LN cells was evaluated using the human cytokine array G3 (RayBiotech). 100 μl of cell culture medium was incubated with the array antibody slide, following the manufacturer's instructions. Signals were detected using Streptavidin and scanned to measure spot intensity. We compared the spot intensity of two pairs of cells: (1) MDA-MB-231LN/Ctrl and /AF1q and (2) /shCtrl and /shAF1q. The relative amount of each cytokine or growth factor is presented as fold change of spot intensity.

### Western blots

Growing medium of the cells was exchanged with 0.5% FBS Opti-MEM (Invitrogen) including doxycycline (1 μg/ml) 2 h prior to lysis. To inhibit *Src* kinase activity, cells were treated with 0.1 μM PP1 (Sigma-Aldrich) for 24 h prior to lysis. Cells were lysed directly in M-PER lysis buffer with the Halt protease inhibitor cocktail and the Halt phosphatase inhibitor cocktail (Pierce) according to the manufacturer's instructions. Cell lysates were separated by electrophoresis on a 4–12% SDS-PAGE gradient gel and blotted on to PVDF membranes. Anti-PDGFR-β, pPDGFR-β, *Src*, STAT3, pYSTAT3 and β-actin monoclonal antibodies were purchased from Cell Signaling Technology and anti-AF1q, p*Src* (pTyr426) and PDGF-B monoclonal antibodies were purchased from Abcam. After incubation with the appropriate antibody, an enhanced chemiluminescence system (Denville) was used for developing blots. Western blot band quantifications were performed with ImageJ analysis software (NIH).

### RNA extraction and qPCR analysis

Total RNA extracted with the mirVana miRNA isolation kit (Invitrogen) was reverse transcribed using high-capacity cDNA reverse transcription kits (Applied Biosystems) according to the manufacturer's instructions. PCR amplification of cDNA encoding PDGF-B was carried out on an ABI 7500 System using TaqMan universal PCR master mix (Applied Biosystems). The following primers were used to amplify PDGF-BB: sense: 5′- TCTCTGCTGCTACCTGCGT-3′, antisense: 5′- CAAAGGAGCGGATCGAGTGG-3′ and HPRT1 (internal control); sense: 5′- CAGAGGG CTACAATGTGATGGC-3′, antisense: 5′- GCTGAGGA TTTGGAAAGGGTG-3′. All samples were run in triplicate and Ct values of internal control and PDGF-B were determined. The relative expression for the target gene was given by 2^−ΔΔCt^.

### PDGF-B ELISA

MDA-MB-231LN cells were plated into 6-well plates at a density of 5×10^5^ cells per well in 0.5% FBS Opti-MEM for 24 h. Supernatants were harvested and PDGF-B levels were measured using a PDGF-B ELISA kit (R&D Systems) according to the manufacturer's instructions.

### PDGF-B promoter assay

The PDGF-B promoter fragment (−1,220/−25 bp from the transcriptional start site; Chr22:39619685-39636914) was PCR-amplified from the genomic DNA of the MDA-MB-231LN cell line using these primers: sense: 5′- CTCGAGAATCCATGGCACAGACCAGC – 3′, antisense: 5′ - AAGTTTTTGGCATCGTGCGTGACAAT - 3′. The resulting PCR product (−1,220/−25) was digested with XhoI and HindIII and ligated into pGL4 luciferase reporter vectors (Promega), resulting in the PDGF-B luciferase promoter construct. Dual-luciferase reporter assays were performed with 1×10^4^ cells plated in each well of a 96-well plate. The next day, the reporter (100 ng) and *Renilla* plasmid (10 ng, Promega) were co-transfected into cells with lipofectamine 2000 (Invitrogen). After 48 h, cells were washed with phosphate-buffered saline (PBS) and assayed with the Dual-luciferase reporter assay system (Promega) according to the manufacturer's instructions. Luciferase activity was determined using the Synergy H1 multi-mode reader (Biotek). Quantitation of the luminescent signal from the reporter plasmid was normalized by quantitation of the luminescent signal from *Renilla*.

### Collection of conditioned medium

When cells reached 70-80% confluency, the medium was changed to 0.5% FBS Opti-MEM, treated with doxycycline (1 μg/ml) for 24 h, and then the culture medium was collected. The collected supernatant was centrifuged for 5 min at 20,000 g at 4°C and used in other experiments as conditioned medium (CM).

### Wound healing and invasion assay

MDA-MB-231LN cells from each group were seeded in 6-well plates at a density of 1×10^6^ cells per well. A 1-mm-wide linear scratch (the wound zone) was established across the center of each well with a micropipette tip and washed three times with medium. We used TScratch software (http://www.cse-lab.ethz.ch/index.php?&option=com_content&view=article&id=363) to evaluate migration rate. We photographed the well under the microscope at the desired time point, and analyzed the open image area using TScratch. The migration rate was calculated as the open image area at 0 h divided by the open image area at 16 h.

For the invasion assay, we used the Fluoroblock HTS insert system (BD Biosciences). 100 μl of matrigel (200 μg/ml) was overlaid into inserts and incubated for 2 h at 37°C. The lower side chambers were filled with 750 μL of culture medium. The cells (2.5×10^4^ cells per insert) were seeded in the upper side chambers with the appropriate CM. For neutralization, anti-PDGF-BB (5 μg/ml, Cell Signaling) or isotype IgG (5 μg/ml, Sigma-Aldrich) was used. For inhibition of PDGFR, MDA-MB-231LN/AF1q cells were treated with imatinib (0.5 μM). Cells were allowed to migrate 24 h and non-migrated cells were removed with cotton swabs. Migrated cells were fixed with ice-cold methanol and stained with calcein AM (4 μg/ml, Invitrogen) for 1 h. Fluorescence intensity was measured with the Synergy H1 multi-mode reader. Experiments were repeated three times independently.

### Electrophoretic mobility shift assay (EMSA)

Nuclear extracts were prepared using the NE-PER™ Nuclear and Cytoplasmic Extraction Kit (Thermo Scientific) according to the manufacturer's instructions. The reaction mixtures (10 μl) contained 5 μg of nuclear extract, 1x binding buffer, 50 ng of poly (dI-dC) and 20 ng of Alexa 488-labeled STAT3 hSIE binding region probe (5′–CATTTCCCGTAAATC-3′, IDT), incubated for 30 min at room temperature. Reaction mixtures containing no nuclear extract were also incubated with labeled probes as negative control samples. Each sample was loaded onto a 6% polyacrylamide gel in 0.5x Tris/Borate/EDTA (TBE) buffer. For the antibody supershift analysis, 1 μg of anti-STAT3 antibody (Cell Signaling) was added to nuclear extracts of MDA-MB-231LN/AF1q cells prior to incubation with the Alexa-488 labeled probe for 30 min. In the competition analysis, 100-fold molar excess of the unlabeled double-strand oligonucleotide was added to the reaction mixture prior to the addition of labeled probe. The reaction mixtures were resolved by electrophoresis using 6% DNA retardation gel (Invitrogen). Samples were electrophoresed until the dye had reached 1 inch from the bottom of the gel and then scanned with the PharosFX Plus (Bio-Rad).

### Tumor growth assay

We performed xenograft transplants to measure tumorigenicity and metastasis *in vivo*, according to the protocol approved by the University of Louisville Institutional Animal Care and Use Committee. The tumor growth assay was performed as previously described [26]. Briefly, MDA-MB-231LN cells labeled with firefly luciferase were diluted with PBS to a concentration of 1×10^7^ cells/ml and 100 μl of cell suspension (1×10^6^ cells) were injected into abdominal mammary fat pads of 6-week old NSG female mice (5 mice/group). For imaging, mice were injected intraperitoneally with a Luciferin solution (150 mg/kg; 15 mg/mL in PBS). The Luciferin solution was allowed to distribute for about 5 minutes, and then an image was taken (1-5 min). Mice were imaged weekly for 4 weeks using the IVIS imaging system. Mice were euthanized and necropsied 4 weeks post-cell injection. Mice were fed 200 mg/kg of doxycycline daily to induce AF1q or shAF1q expression.

### Immunohistochemistry

Tissue samples were fixed in 4% paraformaldehyde and embedded in paraffin, and then 4-μm sections were prepared. Sections were de-waxed and steam pre-treated in Tris/EDTA buffer (DAKO). Endogenous peroxidase activity was quenched by incubation in 3% hydrogen peroxide in PBS. For blocking steps, Avidin (Sigma-Aldrich), Biotin (Sigma-Aldrich) in PBS, and a Super Block (ID Labs Biotechnology) were used. Rabbit monoclonal pYSTAT3 antibody in a 1:200 dilution was incubated at 4°C overnight. IHC detection was performed with the IDetect Super Stain System HRP (ID Labs Biotechnology). Specific signals were amplified using 3-amino-9-ethylcarbazole (ID Labs Biotechnology) under visual control, followed by counterstaining with hematoxylin.

## SUPPLEMENTARY FIGURES


