# Supplementary material for: MLLT11/AF1q boosts oncogenic STAT3 activity through Src-PDGFR tyrosine kinase signaling
Source: Oncotarget. 2016 Jun 1;7(28):43960–73. doi: 10.18632/oncotarget.9759 (PMC5190071; doi:10.18632/oncotarget.9759)
Supplement: Supplementary file 1 [file oncotarget-07-43960-s001.pdf]

# MLLT11/AF1q boosts oncogenic STAT3 activity through *Src*-PDGFR tyrosine kinase signaling

## SUPPLEMENTARY FIGURES

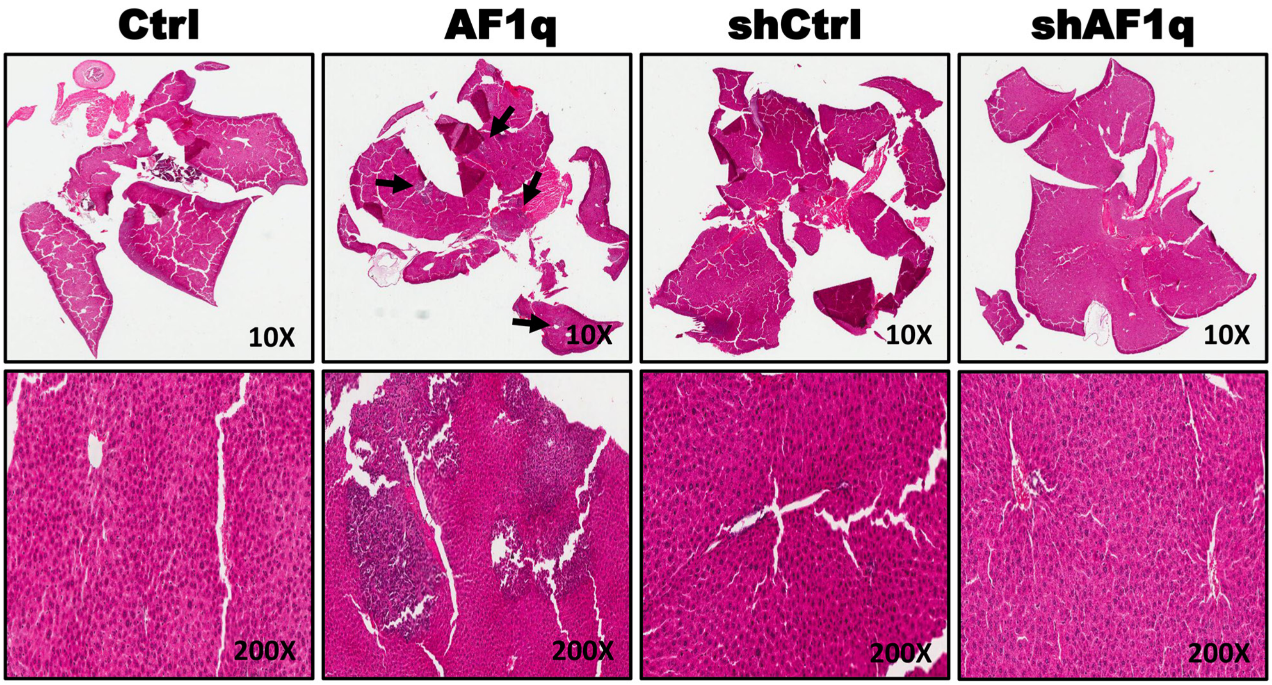


**Supplementary Figure S1: Representative H&E staining of livers as indicated. Black arrows indicate the metastases.**

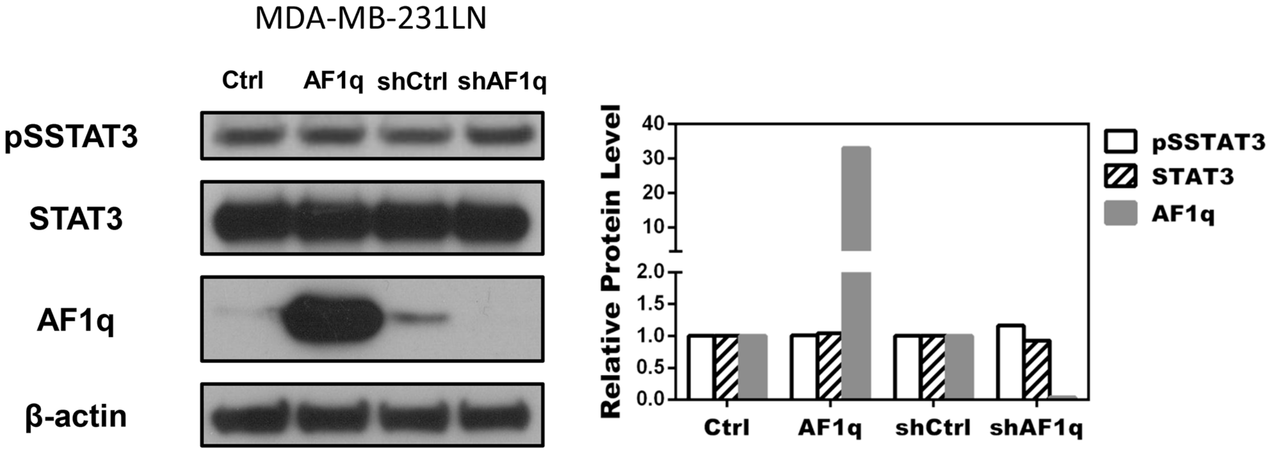


**Supplementary Figure S2: Phosphorylation of serine 727 of STAT3 (pSSTAT3) was not changed by expression of AF1q in MDA-MB-231LN cells.** Band density was determined by ImageJ analysis software.
